# From invisibility to care: A FEBRASGO call to action on violence against women

**DOI:** 10.61622/rbgo/2026rbgoedt1

**Published:** 2026-02-12

**Authors:** Agnaldo Lopes da Silva, Lia Cruz Vaz da Costa Damasio, Maria Auxiliadora Budib, Marcelo Luís Steiner, Roseli Mieko Yamamoto Nomura, Alberto Trapani, Olímpio Barbosa de Moraes, Sérgio Podgaec, Hilka Flavia Barra do Espírito Santo Alves Pereira, Marcos Felipe Silva de Sá, Maria Celeste Osório Wender

**Affiliations:** 1 Universidade Federal de Minas Gerais Belo Horizonte MG Brazil Universidade Federal de Minas Gerais, Belo Horizonte, MG, Brazil.; 2 Universidade Federal do Piauí Teresina PI Brazil Universidade Federal do Piauí, Teresina, PI, Brazil.; 3 Universidade Federal de Mato Grosso do Sul Pioneiros MS Brazil Universidade Federal de Mato Grosso do Sul, Pioneiros, MS, Brazil.; 4 Centro Universitário Faculdade de Medicina do ABC Santo André SP Brazil Centro Universitário Faculdade de Medicina do ABC, Santo André, SP, Brazil.; 5 Universidade Federal de São Paulo São Paulo SP Brazil Universidade Federal de São Paulo, São Paulo, SP, Brazil.; 6 Universidade Federal de Santa Catarina Florianópolis SC Brazil Universidade Federal de Santa Catarina, Florianópolis, SC, Brazil.; 7 Universidade de Pernambuco Recife PE Brazil Universidade de Pernambuco, Recife, PE, Brazil.; 8 Universidade de São Paulo São Paulo SP Brazil Universidade de São Paulo, São Paulo, SP, Brazil.; 9 Universidade Federal do Amazonas Manaus AM Brazil Universidade Federal do Amazonas, Manaus, AM, Brazil.; 10 Universidade de São Paulo Ribeirão Preto SP Brazil Universidade de São Paulo, Ribeirão Preto, SP, Brazil.; 11 Universidade Federal do Rio Grande do Sul Porto Alegre RS Brazil Universidade Federal do Rio Grande do Sul, Porto Alegre, RS, Brazil.

Violence against women remains one of the most serious and persistent challenges in Brazil, with profound consequences that extend far beyond the sphere of public security. Physical, sexual, psychological, and economic violence affect millions of women every year, often repeatedly and within intimate relationships. Every year, hundreds of millions of women worldwide experience violence — most often invisible, recurrent, and underreported. Despite legal advances and the expansion of specialized services, violence against women continues to be largely invisible in everyday clinical practice.

Health systems are uniquely positioned to interrupt this invisibility. In particular, gynecology and obstetrics occupy a central role, given their sustained contact with women throughout the life course. Pregnancy, childbirth, the postpartum period, and routine gynecologic care represent moments of intense interaction between women and health services, frequently marked by trust and continuity. These encounters offer critical opportunities to recognize violence, provide initial clinical support, and activate appropriate pathways of care.

This editorial argues that violence against women in Brazil must be understood as a core women's health issue and that confronting it is a responsibility of the whole of gynecology and obstetrics. Silence in clinical practice is not neutral; it contributes to missed opportunities for protection, prevention, and care.

## The magnitude of violence against women in Brazil

National data consistently demonstrate the scale and severity of violence against women in Brazil. Reports from the Brazilian Public Security Forum indicate that sexual violence has reached historically high levels. According to the *Brazilian Yearbook of Public Security 2024*, 74,930 cases of rape were officially recorded in 2023, representing the highest number ever documented in the country and corresponding to approximately one reported rape every six minutes nationwide.^(1)^ These figures almost certainly underestimate the true burden, given the well-documented barriers to reporting sexual violence.

Particularly alarming is the age profile of victims. Data from the same report show that 61.4% of reported victims of sexual violence are children aged 0–13 years, underscoring the depth of vulnerability associated with sexual violence in Brazil and its intersection with gender, age, and social inequality.^(1)^

Lethal violence represents the most extreme manifestation of this continuum. Recent national reports show that Brazil has recorded more than one thousand feminicides annually since the crime was formally classified, with recent years reaching the highest levels observed since the introduction of the legal definition.^(2)^ On average, several women are killed every day because of their gender. Importantly, feminicide rarely occurs in isolation; it is commonly preceded by repeated episodes of physical, sexual, or psychological violence.

Beyond lethal outcomes, population-based surveys reveal that a substantial proportion of Brazilian women experience non-lethal forms of violence at some point in their lives, including threats, assaults, sexual coercion, and emotional abuse.^(3)^ Taken together, these data underscore that violence against women is not a marginal phenomenon, but a widespread and structural problem with direct implications for health care. These figures alone justify why violence against women cannot be viewed as a peripheral concern within clinical practice.

## Who perpetrates violence and where it occurs

Understanding the profile of perpetrators is essential for clinical recognition. Brazilian studies consistently show that most violent acts against women are committed by men known to the victim, particularly current or former intimate partners, and that the home is the most common setting for abuse.^(1,3)^ This intimate context explains why violence often remains hidden and why health professionals may be among the few external actors with sustained access to women at risk. In cases of feminicide, analyses indicate that partners or ex-partners are frequently responsible and that previous episodes of violence are commonly documented.^(2)^ These findings reinforce that lethal violence is often the endpoint of a trajectory of escalating abuse—one that may be detectable in clinical settings if professionals are attentive to warning signs.

## Violence against women as a women's health issue

Violence against women is a well-established determinant of adverse health outcomes. Brazilian and international studies associate exposure to violence with higher rates of sexually transmitted infections, unintended pregnancy, unsafe abortion, chronic pelvic pain, sexual dysfunction, depression, anxiety, and post-traumatic stress disorder.^(4-6)^ These consequences may persist long after the violent event itself, shaping women's health trajectories over time. Pregnancy does not protect women from violence. On the contrary, evidence indicates that violence may begin or intensify during pregnancy and the postpartum period.^(7)^ Violence during pregnancy has been associated with delayed prenatal care, poorer adherence to medical recommendations, obstetric complications, and adverse neonatal outcomes. These findings place obstetric care at the center of efforts to identify women exposed to violence.

## Why Gynecology and Obstetrics are central

Gynecologists and obstetricians are not peripheral actors in the response to violence against women; they are frontline professionals. The longitudinal nature of gynecologic and obstetric care, the frequency of consultations, and the intimate focus of clinical encounters create conditions for trust that are rarely present in other areas of medicine. However, disclosure of violence is uncommon unless clinicians create explicit opportunities for it. Studies show that without intentional and sensitive inquiry, most cases remain undisclosed.^(7-9)^ Silence should not be interpreted as absence of violence, but rather as a reflection of fear, stigma, and lack of perceived safety within clinical environments. Recognizing violence requires a shift in clinical perspective. Gynecologists and obstetricians must consider violence as a possible underlying factor in gynecologic complaints, obstetric complications, mental health symptoms, and recurrent use of health services. This awareness is a fundamental component of high-quality women's health care.

## Identification in clinical practice

Identification of violence in health care settings is often perceived as complex, yet it can begin with simple, respectful questions asked in a private and confidential environment. Validated tools such as the Abuse Assessment Screen (AAS), the Woman Abuse Screening Tool (WAST), and HITS have demonstrated feasibility and acceptability in gynecologic and obstetric care and can facilitate structured, nonjudgmental inquiry.^(9,10)^ Importantly, identification does not imply investigation, confrontation, or automatic legal action. The primary goals are to recognize the woman's experience, assess immediate safety, and provide information about available resources. For many women, being asked and believed by a trusted physician represents a critical step toward breaking isolation.

## Clinical response and support

The response following disclosure is as important as identification itself. Women who disclose violence require a response characterized by respect, validation, and clarity. Minimizing the experience, expressing disbelief, or avoiding the topic may reinforce silence and discourage future help-seeking. Gynecologists and obstetricians are not expected to resolve complex social or legal situations alone. Their responsibilities include documenting findings appropriately, addressing immediate clinical needs, informing women about available services, and facilitating referral when indicated. Clear institutional protocols and referral pathways are essential to support clinicians in this role.

## Barriers and the need for institutional responsibility

Health professionals in Brazil face real barriers in addressing violence against women, including limited consultation time, lack of privacy, insufficient training, and fragile intersectoral networks.^(10,11)^ These challenges are particularly pronounced in public health services, which care for populations at higher risk of violence. Recognizing these barriers underscores the need for institutional, rather than purely individual, solutions. Evidence from Brazilian system-level interventions shows that organizational strategies —combining professional training, leadership engagement, standardized documentation, and referral networks—can improve identification and response to violence.^(12)^

## Professional leadership and the role of FEBRASGO

Professional leadership by scientific societies extends beyond guideline development and includes sustained engagement with education, ethics, advocacy, and professional protection. In Brazil, the Brazilian Federation of Gynecology and Obstetrics Societies (FEBRASGO) plays a central role in shaping clinical standards, professional culture, and public discourse in women's health. FEBRASGO has been instrumental in advancing the recognition of violence against women as a core women's health issue, promoting this agenda through scientific sessions, educational activities, and awareness campaigns integrated into the Brazilian Congress of Gynecology and Obstetrics and across state-level congresses. These initiatives are complemented by national campaigns, institutional communications, and programs aimed at increasing clinical awareness, reducing stigma, and supporting gynecologists and obstetricians in identifying and responding to violence as part of routine care. A cornerstone of these efforts is the national "#*EuVejoVocê*" ("I See You") (Available from: https://www.febrasgo.org.br/images/FEBRASGO_Cartilha-EuVejoVoce_V5.pdf) campaign, launched in 2025 in partnership with state societies, conceived as a sustained movement to confront violence across all stages of life by challenging harmful norms and empowering clinicians to support women in situations of vulnerability.

Beyond this specific agenda, FEBRASGO consistently incorporates scientific updates, ethical reflection, legal aspects of practice, and issues related to professional valorization into its educational activities. The Federation develops and disseminates clinical guidelines, consensus documents, and peer-reviewed scientific publications that translate evidence into daily clinical decision-making. It also provides continuous institutional guidance to its members through regular communications addressing ethical dilemmas, legal responsibilities, professional defense, and real-life clinical challenges faced in gynecologic and obstetric practice.

At the policy level, FEBRASGO actively engages with parliamentarians, legislators, and health authorities, advocating for the incorporation, recognition, and fair valuation of gynecologic and obstetric procedures within health systems, as well as for regulatory frameworks that protect both women and professionals. In collaboration with other national medical organizations, the Federation works to strengthen professional autonomy, improve working conditions, and enhance societal recognition of gynecologists and obstetricians. Across all these actions, FEBRASGO reaffirms its dual and inseparable commitment: to stand with women at all stages of life and to stand with Brazilian gynecologists and obstetricians throughout their professional journey—expressed in its guiding message that gynecologists and obstetricians are always with women, and that FEBRASGO is always with Brazilian gynecologists and obstetricians ("*Sempre com Você*") (Available from: https://www.febrasgo.org.br/pt/noticias/item/2249-febrasgo-lanca-campanha-sempre-com-voce https://www.febrasgo.org.br/pt/noticias/item/2249-febrasgo-lanca-campanha-sempre-com-voce).

## Breaking invisibility: a professional responsibility

Ultimately, moving from invisibility to care requires an explicit professional stance. For gynecology and obstetrics, recognizing and responding to violence against women is not an additional task, nor a matter of individual sensitivity, but an integral part of ethical and high-quality women's health care. In a country where sexual violence, domestic abuse, and feminicide remain frequent, silence within clinical practice perpetuates harm. By identifying violence, responding appropriately, and engaging with coordinated networks of care, gynecologists and obstetricians reaffirm their central role in protecting women's health, dignity, and lives.
